# Mapping Resection Progress by Tool-Tip Tracking during Brain Tumor Surgery for Real-Time Estimation of Residual Tumor

**DOI:** 10.3390/cancers15030825

**Published:** 2023-01-29

**Authors:** Parikshit Juvekar, Erickson Torio, Wenya Linda Bi, Dhiego Chaves De Almeida Bastos, Alexandra J. Golby, Sarah F. Frisken

**Affiliations:** 1 Department of Neurosurgery, Brigham and Women’s Hospital, Boston, MA 02115, USA; 2 Harvard Medical School, Boston, MA 02115, USA; 3 Department of Radiology, Brigham and Women’s Hospital, Boston, MA 02115, USA

**Keywords:** resection mapping, residual tumor, tumor resection, neurosurgical oncology, image guided neurosurgery, brain shift, intraoperative MRI, intraoperative ultrasound, global surgery, open source

## Abstract

**Simple Summary:**

Surgical resection continues to be the primary therapeutic strategy in neurosurgical oncology. Computerized cranial neuronavigation based on preoperative imaging can offer precision guidance during early tumor resection but loses validity as the procedure progresses with tissue removal and shifting. Modalities such as intraoperative MRI (iMRI) and intraoperative ultrasound (iUS) can restore image guidance to maximize the extent of resection but present challenges in terms of temporal and spatial resolution, respectively. Our study leverages an untapped data stream from clinical neuronavigation systems to track time-stamped tool-tip positions of surgical instruments. This enables the mapping of resection progress with temporal and spatial accuracy for the real-time estimation of residual tumors. By itself, our technique could serve as an alternative to iMRI for resource-limited regions of the world and as an educational training and evaluation tool. It could also be combined with other intraoperative imaging modalities, such as iUS, to more accurately model and compensate for brain shift.

**Abstract:**

Surgical resection continues to be the primary initial therapeutic strategy in the treatment of patients with brain tumors. Computerized cranial neuronavigation based on preoperative imaging offers precision guidance during craniotomy and early tumor resection but progressively loses validity with brain shift. Intraoperative MRI (iMRI) and intraoperative ultrasound (iUS) can update the imaging used for guidance and navigation but are limited in terms of temporal and spatial resolution, respectively. We present a system that uses time-stamped tool-tip positions of surgical instruments to generate a map of resection progress with high spatial and temporal accuracy. We evaluate this system and present results from 80 cranial tumor resections. Regions of the preoperative tumor segmentation that are covered by the resection map (True Positive Tracking) and regions of the preoperative tumor segmentation not covered by the resection map (True Negative Tracking) are determined for each case. We compare True Negative Tracking, which estimates the residual tumor, with the actual residual tumor identified using iMRI. We discuss factors that can cause False Positive Tracking and False Negative Tracking, which underestimate and overestimate the residual tumor, respectively. Our method provides good estimates of the residual tumor when there is minimal brain shift, and line-of-sight is maintained. When these conditions are not met, surgeons report that it is still useful for identifying regions of potential residual.

## 1. Introduction

Surgical resection continues to be the primary initial therapeutic strategy in the treatment of most brain tumors, with the extent of tumor resection strongly correlated with prognosis and overall survival [1,2,3]. Attempts to maximize the extent of resection while preserving the neurological function by avoiding injury to adjacent healthy tissue have inspired the innovation of tools to support resection with high spatial accuracy.

Computerized cranial neuronavigation is a powerful adjunct that can facilitate maximal safe resection of brain tumors on the basis of preoperative MRI or CT. However, cranial neuronavigation loses validity as the surgery progresses because of brain shift and non-linear deformation of the resection cavity, making the information available to the surgeon less useful [4,5].

Intraoperative MRI (iMRI) can mitigate the declining accuracy of cranial neuronavigation by providing updated images of the resection status that are comparable in spatial resolution to preoperative MRI [6,7]. However, iMRI mandates significant infrastructural investments, dedicated personnel, and high operating costs, which are not feasible for most healthcare centers [8]. Closed-bore configurations with higher field strengths up to 3 Tesla (T) require the patient to either be moved to the MRI magnet or have the MRI magnet moved over the patient on the operating room table. This disrupts the surgical workflow and lengthens the procedure time, thereby limiting the number of timepoints at which iMRI can be leveraged during each surgery and the number of patients who can benefit from the technology, even in centers equipped with this technology [4].

Intraoperative ultrasound (iUS) has recently re-gained popularity as a low-cost, portable, and efficient method to serially assess the extent of resection intraoperatively with minimal disruption to the surgical workflow [4]. One limitation of iUS is its limited signal-to-noise ratio (SNR) and inability to discern tissue contrasts compared to the gold standard of iMRI for determining the extent of resection [9]. In addition, iUS is usually acquired in oblique planes–different from the usual axial, coronal, and sagittal views neurosurgeons are familiar with–making these images difficult to interpret, especially while the surgical team is cognitively and visually overloaded with other tasks.

Currently, even with contemporary surgical adjuncts, a surgeon must maintain a mental map of resected regions to determine areas of residual tumor. This can be particularly challenging with long surgical durations, changes in patient positioning, multiple angles of approach, contribution to resection by multiple surgeons, irregularly shaped lesions, and tumors, especially gliomas, that are often indistinguishable from healthy brain tissue. Thus, during tumor resections, surgeons are still often unsure about whether the tumor has been completely resected and, if not, where the residual tumor may be. To bridge this unmet clinical need, we proposed mapping the progress of the surgical resection by continuously recording the tool-tip positions of standard surgical instruments tracked using standard-of-care neuronavigation. Our resection map aims to reduce this cognitive burden on the surgeon by providing real-time visualization of resection status and potential areas of residual tumor in inconspicuous regions of the resection cavity [10].

To validate the logging of tool-tip positions as a realistic alternative for residual tumor estimation, we first evaluated its accuracy for a tracked Cavitron ultrasonic surgical aspirator or CUSA (Integra LifeSciences, Princeton, NJ, USA) in 18 gelatin phantoms. We used a signed distance field [11] to represent the resection map and achieved a sub-millimeter resolution and real-time processing speed. We found the average coverage over the preoperative lesion segmentation to be 97.5% and the average overlap with the segmented resection cavity to be 94.7%. We then assessed the feasibility of ensuring minimal interruption to the surgical flow in 15 clinical cases with promising results [10].

In this publication, we describe the further optimization of our workflow and test its clinical promise in predicting residual tumors in a large patient cohort. We also identify nuanced considerations and suggest workflow refinements to encourage successful clinical translation to surgical practice. By validating our work against iMRI, we also explore its potential as a low-cost alternative to maximize the extent of resection for the vast majority of patients in the world who do not have access to iMRI.

## 2. Materials and Methods

### 2.1. Patient Selection

**Figure 1:**
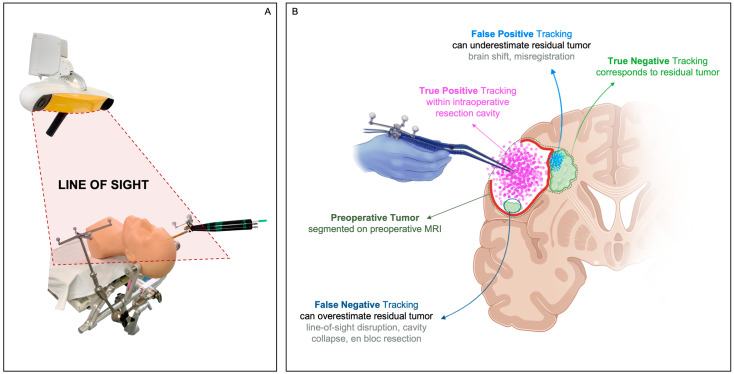
Panel **A**—Line-of-sight. Panel **B**—True Positive Tracking should ideally correspond to the intraoperative resection cavity estimated on iMRI. False Positive Tracking due to brain shift or misregistration can underestimate residual tumor. False Negative Tracking due to line-of-sight disruption, cavity collapse, en bloc resection can overestimate residual tumor. True Negative Tracking should ideally correspond to the residual tumor estimated on iMRI.

Eighty patients undergoing craniotomy for cranial tumor resection between March 2019 and November 2022 in the Advanced Multimodality Image Guided Operating Suite (AMIGO) [12] at Brigham and Women’s Hospital (Boston, MA, USA) prospectively consented to intraoperative data collection using the neuronavigation system. From this pool, 36 patients were excluded during postoperative analysis primarily for low tracking point density, which was attributed to line-of-sight disruption (Figure 1 Panel A), as described in Section 4.2, producing a final cohort of 44 resections (from 42 patients). Cases 25 and 26 correspond to the same patient (Appendix A), in which the surgeon divided the resection plan into two distinct regions (with some overlap)—an enhancing component and a T2 signal abnormality. Hence, this patient’s surgery was analyzed as two separate cases within our cohort from the standpoint of mapping resection progress over these two preoperative lesion segmentations. Additionally, Cases 6 and 17 also correspond to the same patient who returned due to the recurrence of their tumor.

$$\begin{array}{l} Predicted\;Extent\;of\;Residual\;Tumor\\ =\;True\;Negative\;Tracking\;+\;False\;Negative\;Tracking\\ =\;Preoperative\;Tumor\;Segmentation\text{—}(True\;Positive\;Tracking\;+\;False\;Positive\;Tracking) \end{array}$$

### 2.2. Preoperative Planning

Preoperative surgical planning was performed as part of the standard of care using the Brainlab Elements Planning Software platform (Brainlab AG, Munich, Germany). Clinically relevant MRI series were co-registered. The cerebrum was segmented automatically on the clinical neuronavigation platform. The lesions targeted for resection and any resection cavities from previous tumor resections were manually segmented by clinical experts in brain tumor segmentation. For contrast-enhancing lesions, a 3D 1 mm isovoxel T1-weighted series with gadolinium was selected as the basis for manual segmentation. For non-enhancing lesions, a 3D 1 mm isovoxel T2 SPACE [13] series, a 2D 2 mm BLADE [14] series, or a 3D 1 mm isovoxel MP2RAGE [15] series was selected as the basis for tumor segmentation. Additionally, whenever applicable, segmentations of blood oxygen level-dependent (BOLD) functional MRI (fMRI) activations and white matter tractography derived from diffusion MRI (dMRI) were included in the preoperative plan to inform tumor resection.

### 2.3. Neuronavigation and Instrument Tracking

**Figure 2:**
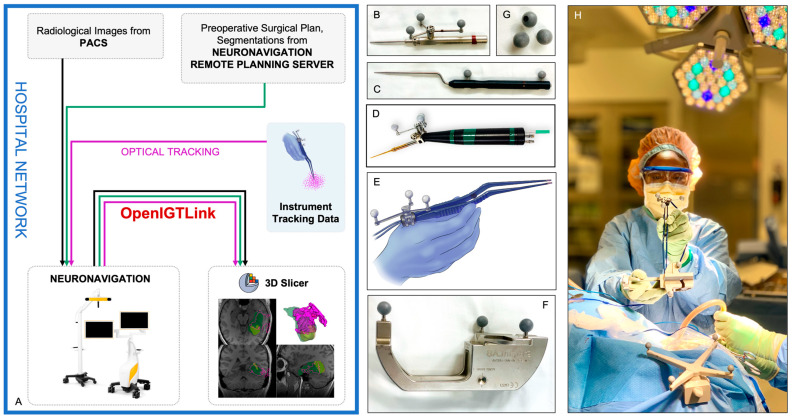
Panel **A**—Schematic of the network connections with the direction of data flow between the various software components used in our technique. Preoperative and intraoperative radiological imaging was downloaded to the neuronavigation system from the hospital picture archiving and communication system (PACS). Segmentations and other preoperative surgical planning objects were retrieved from the remote planning server platform by the neuronavigation system. Tool-tip positions of optically tracked surgical instruments were streamed to the neuronavigation system. All three data types were, in turn, streamed through OpenIGTLink from the clinical neuronavigation platform to the research platform implemented in 3D Slicer. Panel **B**—Brainlab multiple-tip pointer with instrument adapter array. Panel **C**—Pre-calibrated Brainlab cranial pointer with a blunt tip. Panel **D**—Cavitron ultrasonic surgical aspirator (CUSA) with instrument adapter array. Panel **E**—Bipolar forceps with instrument adapter array. Panel **F**—Brainlab instrument calibration matrix. Panel **G**—Reflective marker spheres for optical neuronavigation. Panel **H**—Surgical team member performing the calibration process using the instrument calibration matrix for bipolar forceps mounted with a size ‘M’ instrument adapter array.

The Brainlab Curve Dual Display system (Brainlab AG, Munich, Germany) was used for intraoperative optical neuronavigation. A wired network connection was provided to access radiological imaging from the hospital picture archiving and communication system (PACS) for retrieving preoperative surgical plans from the Brainlab remote server as well as establishing communication through the OpenIGTLink [16] interface to the open-source data visualization platform 3D Slicer [17] (Figure 2 Panel A). Image-to-patient surface registration was performed using the registration module on the neuronavigation system.

To track the progression of resection, size ‘M’/‘ML’ instrument adapter arrays from Brainlab affixed to their corresponding adapter clamps were mounted on either a bipolar forceps (Figure 2 Panel E), a CUSA (Figure 2 Panel D) or a Brainlab multiple-tip pointer (Figure 2 Panel B) and calibrated using the Brainlab Instrument Calibration Matrix (Figure 2 Panel F and Panel H). The orientation of these instrument adapter arrays was decided in consultation with the surgeon to maintain maximal line-of-sight, taking into account the angles of surgical approach and the positions of the surgical team members relative to the optical infrared neuronavigation camera.

### 2.4. Data Collection and Visualization of Resection Progress

We established a connection between the Brainlab Curve Dual Display system and a laptop computer running 3D Slicer (version 4.8.1) (www.slicer.org, accessed on 1 January 2019) via OpenIGTLink [16] (Figure 2 Panel A). Upon completion of image-to-patient registration, the preoperative MRI series selected as the basis for tumor segmentation during preoperative surgical planning was queried through OpenIGTLink. This MRI series, now co-registered to the patient space, was loaded in 3D Slicer in the ‘Four-Up’ view configuration—which includes one 3D panel and three 2D panels of orthogonal anatomic planes—axial, coronal, and sagittal. Preoperative segmentations of the tumor, previous resection cavity (in cases of tumor recurrence), and the cerebrum were imported through OpenIGTLink as co-registered 3D volumes with binary voxel intensity values. These segmentations were converted to 3D models using the Grayscale Model Maker module in 3D Slicer. The Models module was then used to set the visibility, color, and opacity of these models in the 3D and 2D panels. A region of interest (ROI) box was created to enclose the tumor and adjacent areas within which the resection progress would be mapped.

Tracking control began with the OpenIGTLink Remote module, which facilitates the import of transforms corresponding to each tracked instrument/adapter array. A custom 3D Slicer module created by Frisken et al. [10] (available for download from the GitHub repository, https://github.com/sarahfrisken/continuous-monitoring-tracking) utilized these transforms to stream time-stamped x, y, z coordinates of tool-tip positions of each tracked instrument to corresponding text files at a rate of 15–30 points/second. Simultaneously, the custom module interpreted this data as a 3D image volume with floating point voxel intensity values to encode tool-tip positions at sub-millimeter resolutions. This 3D volume was visualized both as a 2D overlay on corresponding orthogonal preoperative MRI slices as well as a 3D model updated intermittently (processing time 2–3 s) using the Grayscale Model Maker module. As illustrated in Video S1, the 2D overlay automatically updates (processing time 30–50 milliseconds) as the surgeon moves the instrument in the surgical field. Assuming the instrument is tracked continuously during surgery (i.e., line-of-sight is maintained) and there is limited brain shift, the volume traversed by the instrument tip provides a map of the resection cavity.

### 2.5. Intraoperative Imaging

iMRI was acquired using a 3T wide-bore (70 cm) MRI scanner (Magnetom Verio, Siemens Healthineers, Erlangen, Germany) and an 8-channel head coil after the surgeon felt, based on their usual clinical decision-making, that most or all of the targeted tumor had been resected. For all lesions, a 3D 1 mm isovoxel T2 SPACE [13] series, a 3D 1 mm isovoxel FLAIR series, a 2D 2 mm BLADE [14] series, or a 3D 1 mm isovoxel MP2RAGE [15] series were obtained. For contrast-enhancing lesions, a 3D 1 mm isovoxel T1-weighted series with gadolinium was also acquired. The residual tumor, if any, and the intraoperative resection cavity were manually segmented on the neuronavigation system. These new co-registered segmentations were transferred to 3D Slicer using the OpenIGTLink Remote module.

### 2.6. Postoperative Data Analysis

The volume of the preoperative tumor/lesion and the intraoperative residual tumor segmented from iMRI were calculated using 3D Slicer. A segmentation of the cerebrum was used to filter out tool-tip positions outside the brain as tracked surgical instruments enter and leave the surgical field. The remaining points were used to generate a volumetric model of the tracked resection cavity. This volumetric model was represented as a signed distance field [10].

We define regions of the segmented tumor that were covered by the tracked resection cavity as True Positive Tracking (Figure 1 Panel B) and report this as a percentage of the segmented preoperative tumor volume (Appendix A, Table A1). Under ideal circumstances, this percentage will equal the actual amount of tumor resected, where the actual tumor resected is computed as the residual tumor volume (segmented on iMRI) subtracted from the preoperative tumor volume (segmented on preoperative MRI). Poor sampling due to lack of line-of-sight, brain shift, and cavity collapse (for example, in tumors with large cystic components) can all contribute to differences between the tracked resection cavity and the actual resected tumor.

We define regions of the residual tumor identified by iMRI but which were covered by the instrument tool-tip positions as False Positive Tracking (Figure 1 Panel B) and report these as a percentage of the residual tumor volume (Appendix A, Table A1). Ideally, False Positive Tracking should be zero because it would indicate that resection occurred inside the residual tumor. Brain shift and inaccurate tracking calibration can lead to False Positive Tracking. True Negative Tracking denotes the residual tumor estimated by resection progress mapping, which should ideally equal the residual tumor volume segmented on iMRI.

### 2.7. Additional Clinical Descriptors and Metadata

Patient demographics, tumor pathology, and imaging characteristics were prospectively collected and entered into a Research Electronic Data Capture (REDCap) database. Age, sex, race, and pathology were captured from a chart review (Appendix A, Table A2) of the Electronic Medical Record (EMR). For gliomas, the World Health Organization (WHO) grade, MGMT promoter (methylated/unmethylated/partially methylated), IDH mutation status (yes/no), and 1p/19q co-deletion (yes/no) were recorded postoperatively from anatomic pathology, cytogenetics, and oncopanel reports. Radiographic characteristics such as laterality, previous cranial surgery, anatomic compartments, tumor components, eloquence, and brain shift were determined and compared with imaging reports and intraoperative findings. Eloquence was determined anatomically by adjacent cerebral structures with a readily identifiable neurological function in which injury results in disability or in areas with significant functional MRI (fMRI) activations [18]. The opening of ventricles and/or cisterns was determined based on the operative video, operative record, and post-operative imaging. Any disagreements were consolidated with senior authors. Gross total resection was determined postoperatively based on imaging reports. Descriptive statistics are presented in Table 1 and Table 2. Continuous variables were presented as median [interquartile range (IQR)] and mean ± standard deviation (SD) depending on normality based on the Shapiro–Wilk test. Discrete variables were presented as counts and percentages. Statistical analyses (Table 1 and Table 2) were performed using IBM SPSS Statistics version 28.0 for Mac.

## 3. Results

### 3.1. Distribution of Clinical and Surgical Descriptors

In our cohort of 44 cases (resections) from 42 patients, the median age was 46 years, with slight male preponderance congruent with the epidemiology of brain tumors. Forty-four resections were performed on 42 patients, as one patient underwent reoperation for tumor recurrence (Cases 6 and 17 in Appendix A), and another was analyzed as two separate cases due to multiple resection targets for the same patient (Cases 25 and 26 in Appendix A). The predominant pathology was glioma (84.0%), with metastases making up 14.4%. The distribution of lesion laterality was also approximately equal. Most tumors were located in the frontal, temporal and insular regions. A majority of the cases had a past history of prior cranial surgery (65.9%). Factors with the potential to confound the quantitative estimation of residual tumor by resection mapping include previous resection cavity (40.9%), cystic tumor components (36.4%), the intraoperative opening of ventricles (38.6%), and intraoperative opening of the basal cisterns (34.1%).

**Figure 3:**
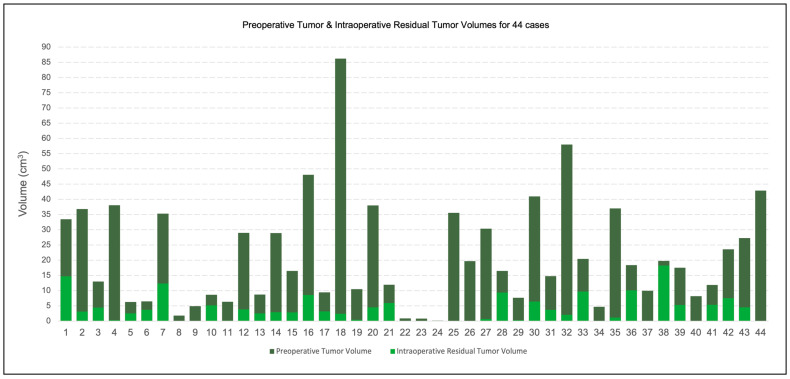
Distribution of preoperative tumor and intraoperative residual tumor volumes for all 44 cases. The light green bars represent the intraoperative residual tumor as a fraction of the underlaid dark green bars, which represent the preoperative tumor. Both bars share the same baseline. For additional details, see Appendix A, Table A1.

Our patient cohort had a wide distribution of preoperative tumor volumes ranging from 0.1 cm^3^ to 86.2 cm^3^, as depicted by the dark green bars in Figure 3. At the time of intraoperative imaging, the amount of residual tumor was significantly reduced, as depicted by the overlapping light green bars in Figure 3. At the time that iMRI was acquired, residual tumor volumes ranged from 0 to 18.2 cm^3^.

### 3.2. Qualitative Results

The accuracy of our resection tracking method depends on many factors, including maintaining good line-of-sight (between the neuronavigation camera and the surgical instrument), calibration accuracy (of the tracked surgical instrument), presence/absence of brain shift, and presence/absence of cystic components in the tumor. Regardless, surgeons using our system provided overwhelmingly positive feedback about its value even when model accuracy was compromised. For one, tracking surgical instruments continuously allowed them to see the location of their instrument relative to preoperative imaging at any time without having to pause and pick up a tracked surgical pointer. Second, the shape of the tracked resection cavity provided them with important cues about potential locations of residual tumor, even when there was substantial brain shift. They found this to be particularly useful in reoperations for recurrent tumors where the shape of the tumor could be quite complex and include multiple concavities.

**Figure 4:**
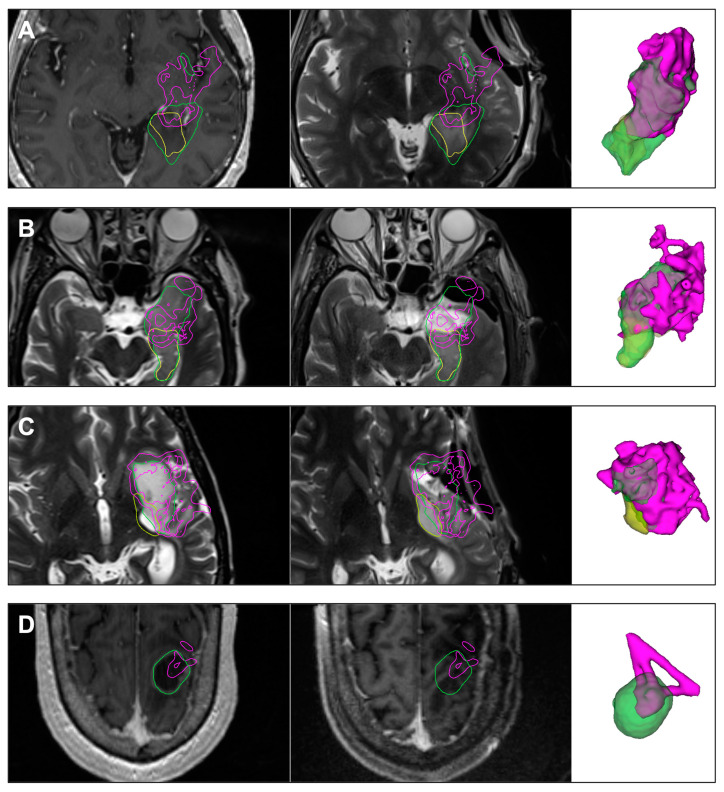
Select clinical cases. Each row shows, left to right, the preoperative MRI, intraoperative MRI, and 3D models of the tumor segmented on preoperative MRI (green), residual tumor segmented on intraoperative MRI (yellow), and the volume traced out by the tracked tool-tip positions of surgical instruments (pink). The contours of the models are also shown in the MRI images in their respective colors. In Panels **A** and **B** (Cases 12 and 36), the tracked resection cavity accurately represents the true resection cavity. In Panel **C** (Case 31), residual tumor volume estimation appears limited due to brain shift, but the residual location is accurate, nevertheless. In Panel **D** (Case 9), the tracked resection cavity does not cover the entire tumor even though there is no residual. This occurred because draining the large cystic component collapsed the resection cavity during surgery.

Our resection tracking method provided an accurate model of the resection cavity when line-of-sight was consistently maintained, and brain shift was minimal. Under these circumstances, the difference between the preoperative tumor segmentation and the tracked resection cavity approximates the residual tumor and could be used to guide further resection. These cases have high True Positive Tracking and low False Negative Tracking. Two such cases are illustrated in Figure 4 Panel A (Case 12) and Figure 4 Panel B (Case 36).

If line-of-sight was frequently interrupted, there were not enough tracked tool-tip positions to provide a good model of the resection cavity. These cases had low True Positive Tracking.

When tumors contain a large cystic component, as illustrated in Figure 4 Panel D (Case 9), draining the cystic component can result in collapse of the resection cavity during surgery. In this case, although the tracked resection cavity is much smaller than the preoperatively segmented tumor volume (low True Positive Tracking), no residual was identified with iMRI.

To further contextualize these scenarios and concepts, we describe Cases 7 and 31 in detail.

#### 3.2.1. Case 7

A 28-year-old right-handed man presented with new-onset seizures. Brain MRI demonstrated a non-enhancing infiltrating mass in the left temporal lobe suspected to be a glioma (reported postoperatively by clinical pathology as WHO Grade 3 Anaplastic Astrocytoma with MGMT promoter methylation). Upon imaging, no cystic regions were identified within the tumor bulk that could potentially collapse and compound brain shift. He underwent functional MRI (fMRI) and diffusion tensor imaging (DTI) studies preoperatively, demonstrating left-lateralized language dominance and the proximity of language-associated fMRI activations in the superior temporal gyrus within 1 cm of the tumor margin.

He was positioned supine in the operating room with his head turned to the right such that the posterior left temporal region was uppermost in the field. The neuronavigation camera was positioned to the patient’s right at a location the surgical team perceived to be least prone to line-of-sight disruption. Patient-to-image registration was achieved using the clinical neuronavigation system. A connection was established with our custom module within 3D Slicer using the OpenIGTLink communication interface (Figure 2 Panel A). The craniotomy was performed through a curved incision above the left ear. The surgeon selected a Brainlab multiple-tip pointer (Figure 2 Panel B) as one of the primary instruments, and it was affixed with a size ‘M’ instrument adapter array. The instrument was calibrated (Figure 2 Panel H) using the Brainlab instrument calibration matrix (Figure 2 Panel F). The tool-tip positions were streamed to our custom module throughout tumor resection, as in Video S1. The tumor margins adjoined the left lateral ventricle, which resulted in its opening. Despite this, minimal to no brain shift was noted on iUS.

**Figure A1:**
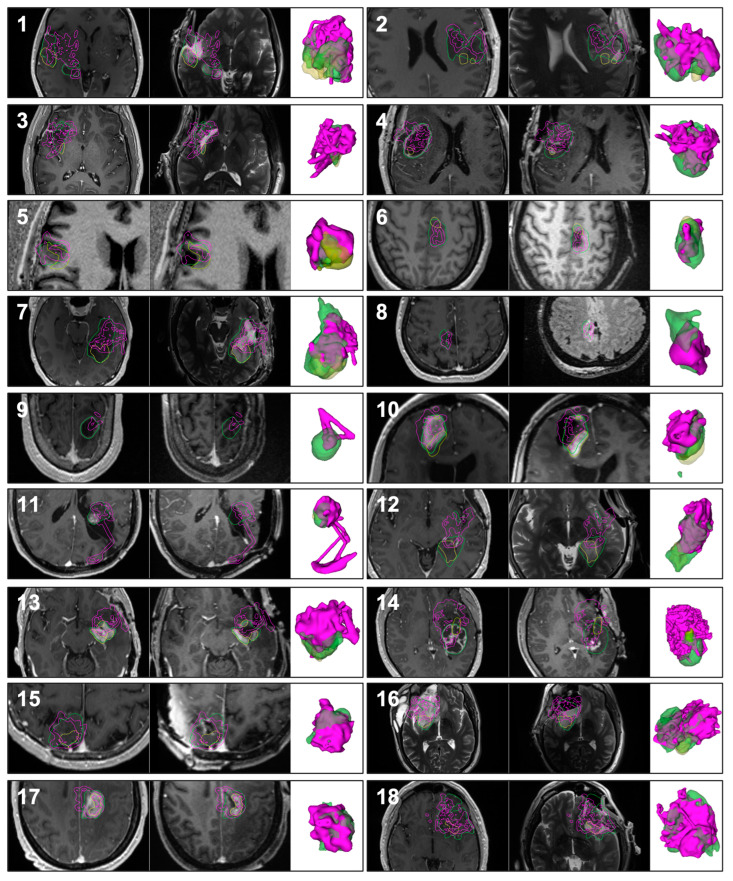

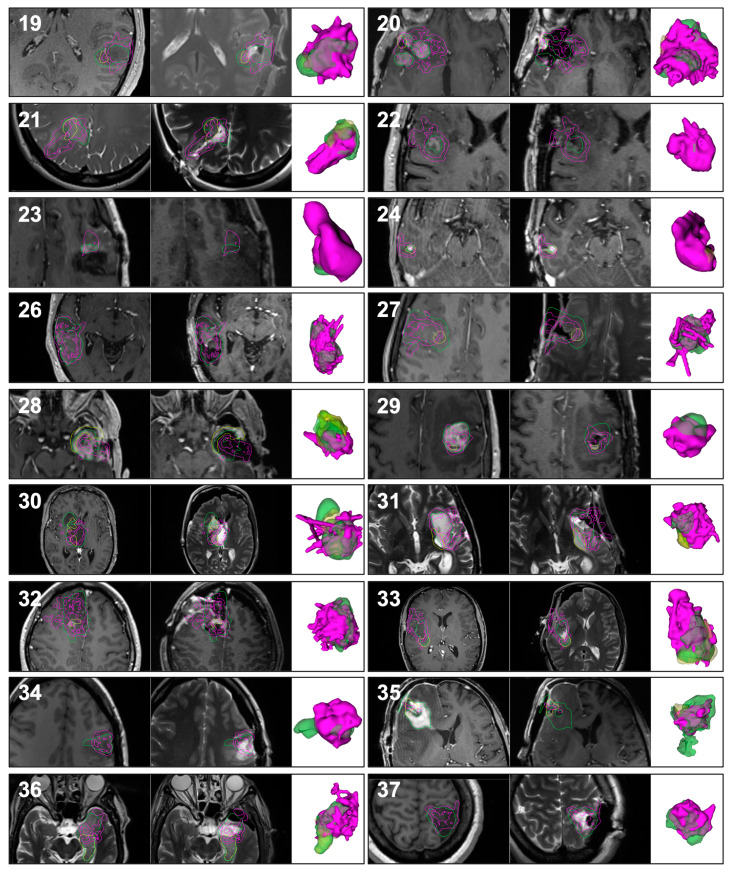

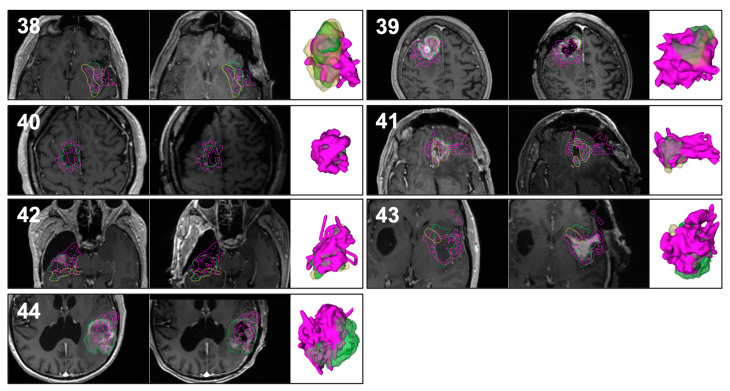
Images of all clinical cases (1 to 44) showing, from left to right and for each case, the preoperative MRI, intraoperative MRI, and 3D models of the tumor segmented on preoperative MRI (green), residual tumor segmented on intraoperative MRI (yellow), and the volume traced out by the tracked tool-tip positions of surgical instruments (pink). The contours of the models are also shown in the MR images by their respective colors. Please note that the orientation of the 3D models bears no relation to the orientation of the MR image slices.

As seen in the panel for Case 7 in Appendix A, Figure A1, and Table A1, our method of resection progress mapping recorded resection of 50.8% (pink) of the 35.3 cm^3^ preoperative tumor segmentation (green), predicting 17.3 cm^3^ to be the estimated volume of the residual tumor (yellow) at the time of iMRI. The panel for Case 7 in Appendix A, Figure A1, also shows that our technique predicted the shape and the location of the residual tumor in the posterior part of the preoperative tumor. The true intraoperative residual tumor subsequently segmented on iMRI was found to be 12.3 cm^3^. Thus, our module overestimated the volume of the residual tumor by 14.3%, which was attributed to False Negative Tracking, and is qualitatively appreciable in the panel for Case 7 in Appendix A, Figure A1.

A similar level of accuracy in predicting the extent and location of residual tumor can also be seen in Cases 12 (Figure 4 Panel A) and 36 (Figure 4 Panel B), where there was minimal to no brain shift.

#### 3.2.2. Case 31

A 45-year-old originally right-handed (adaptively left-handed) man presented for resection of contrast-enhancing recurrence in his left insula status post previous resection of his WHO Grade 2 (now Grade 3) IDH-1 mutant Astrocytoma. On imaging, there were no cystic regions within the bulk of the contrast-enhancing recurrence. However, significant brain shift was anticipated from the previous resection cavity, which communicated with the left lateral ventricle. He underwent fMRI and DTI studies preoperatively, demonstrating left-lateralized language dominance.

He was positioned supine in the operating room with his head turned to the right. The neuronavigation camera was positioned to the patient’s right at a location the surgical team perceived as least prone to line-of-sight disruption. Patient-to-image registration was achieved using the clinical neuronavigation system. A connection was established with our custom module within 3D Slicer using the OpenIGTLink communication interface (Figure 2 Panel A). The prior reverse question-mark incision was utilized to access and extend his previous craniotomy. The surgeon selected the bipolar forceps (Figure 2 Panel E) as one of the primary instruments, and it was affixed with a size ‘M’ instrument adapter array. The instrument was calibrated (Figure 2 Panel H) using the Brainlab instrument calibration matrix (Figure 2 Panel F). The tool-tip positions throughout tumor resection were streamed to our custom module, as in Video S1. During resection, a significant level of brain shift was observed on iUS when opening the ventricle and the cistern.

As seen in Figure 4 Panel C, our method of resection progress mapping recorded a resection of 75.2% (pink) of the 14.7 cm^3^ preoperative tumor segmentation (green), predicting 3.7 cm^3^ to be the estimated volume of the residual tumor (yellow) at the time of iMRI. The figure also shows that our technique correctly predicted the location of the residual tumor along the posteromedial margin of the preoperative tumor and its shape. The true intraoperative residual tumor subsequently segmented on iMRI was also found to be 3.7 cm^3^. Thus, Case 31 illustrates a scenario where our technique could correctly identify the extent and location of the residual tumor even in the presence of significant brain shift.

Another important consideration when qualitatively interpreting Figure 4 Panel C is that the retraction applied during tumor resection is absent during MRI, causing the resection tracking points (pink) to appear falsely over the nearby healthy parenchyma beyond the margin of the preoperatively segmented tumor. This is also seen in Case 13. A similar situation is noted for Cases 1, 12, 14, 27, and 33, depicted in Appendix A, Figure A1, during which the Sylvian fissure was opened and retracted. This discrepancy can be difficult to comprehend without this surgical context, but it was intuitive to the operating surgeon based on their chosen approach.

For qualitative results of all 42 patients, see Appendix A, Figure A1.

### 3.3. Quantitative Results

Measurements of the preoperative tumor, residual tumor (on iMRI), and tracking data (summarized in Table 3, full dataset in Appendix A, Table A1) show that the tracked resection cavity tended to underestimate the actual percentage of tumor resected (and thus overestimate the size of residual tumor), as measured at iMRI. The underestimation of the resected tumor was greater when brain shift and/or a large cystic component was present.

The mean preoperative tumor volume was 21.1 cm^3^, and the median was 16.4 cm^3^, with tumors ranging from less than 0.1 cm^3^ to 86.2 cm^3^. At the time of intraoperative imaging, the mean residual tumor volume was 3.9 cm^3^, and the median was 2.9 cm^3^, with residual volumes ranging from 0 to 18.2 cm^3^. The percent of the lesion resected at the time of intraoperative imaging ranged from 7.7% (a planned surgical biopsy) to 100% with a mean (median) of 79.1% (86.7%).

The average True Positive Tracking (percent of the true resection cavity estimated on iMRI covered by the tracked tool-tip positions) was 61.5%, confirming our observation that the tracked resection cavity tends to underestimate the true resection cavity.

The mean (median) False Negative Tracking (percent of the residual tumor estimated on iMRI covered by the tracked resection cavity) was 16.2% (12.6%) and ranged from 1.5% to 47.9%. Thus, in all cases, even in the presence of brain shift or cystic components, the lack of tracking in a region of the preoperatively segmented tumor provided a possible location of residual tumor. Surgeons using the system noted that this information alone was helpful because it alerted them to regions they should explore further during resection.

## 4. Discussion

We present the largest study of navigation-logged tool-tip positions for surgical instruments tracked during the resection of brain tumors. Our novel technique functions parallel to standard-of-care commercial neuronavigation without disrupting the surgical workflow and utilizes a data stream that is otherwise discarded at the backend. Our solution records tool-tip positions at a rate of 15–30 points/second with sub-millimeter spatial resolution—the highest reported in the literature thus far [10]. Our results demonstrate that this technique provides significant clinical benefit at minimal to no additional cost. It also may shorten the surgical duration through continuous, interruption-free navigation and minimize the number of instances when the surgeon needs to pause and switch instruments to orient themselves.

### 4.1. System Compatibility and Generalizability

We tested our open-source 3D Slicer module by manually downloading and installing it in version 4.8.1 of 3D Slicer [17]—a free, open-source software for medical image visualization, analysis, quantification, segmentation, and registration. OpenIGTLink IF and OpenIGTLink Remote modules [16] are available for automatic installation through the Extension Manager wizard within all versions of 3D Slicer, including 4.8.1. 3D Slicer can be installed on various operating systems, such as Microsoft Windows 7 (and newer), macOS, and a variety of Linux distributions.

We performed our experiments using the Brainlab Curve Dual Display neuronavigation system due to institutional availability. However, it should be possible to replicate this experimental system with other standard-of-care commercial neuronavigation systems, such as the Medtronic StealthStation (with a StealthLink license), in conjunction with the PLUS toolkit (https://plustoolkit.github.io/) [19].

Low-cost neuronavigation platforms under development, such as NousNav [20], offer another promising avenue for the deployment of this technology to lower-resource settings. The software component for NousNav, being a custom open-source fork of 3D Slicer, improves its potential compatibility with our technique and also eliminates the expensive precondition of purchasing interface licenses (e.g., StealthLink) from commercial vendors.

Our method of tracking resection is also agnostic to the users’ preferred manner of visualization. Thus, it can be potentially integrated with augmented reality (AR) and virtual reality (VR) systems which are increasingly being implemented in neurosurgical practice [21].

### 4.2. Line-of-Sight

Maintenance of line-of-sight (Figure 1 Panel A) between the camera sensor of an optical neuronavigation system and the tracked instrument is the most significant challenge to the practical translation of this technique. Interruption of line-of-sight by physical objects and team members can result in False Negative Tracking and, thus, overestimate residual tumor (Figure 1 Panel B). Prior publications [22,23] have reiterated this correlation between line-of-sight and the accuracy of residual tumor estimation.

Our experience has been similar and is reflected by the 36 excluded cases from the early phase of our study as we incrementally refined our workflow with participation from all operating room staff. We recognized the importance of a preoperative discussion with the surgeon to determine potential angles of approach, optimal locations for the optical neuronavigation camera in the operating room, preferred instruments for tracking, and the ideal orientation of the instrument adapter array to ensure maximum visibility. Other commonly overlooked details include the potential positions of the surgical technologist, the instrument tables, and the side to which the assistant microscope oculars are attached. While these efforts significantly improved line-of-sight, completeness of the tool-tip position log, and the accuracy of residual tumor estimation, we did recognize short timeframes during the surgery when ensuring line-of-sight required active adjustment of the camera sensor position by a team member. At times, even when perfect line-of-sight was confirmed, we observed interference from the operating microscope light reflecting off the marker spheres (Figure 2 Panel G), resulting in the instrument tracking array not being perceived by the optical camera sensor.

As these practical challenges were gradually addressed, we noted an upward trend in the quality of our data, and we expect a much lower exclusion rate for cases going forward. Additionally, the use of multiple optical sensors, the augmentation of optical neuronavigation camera systems with automated target tracking, and the substitution/supplementation with electromagnetic neuronavigation warrant further investigation as solutions to some of the enduring issues.

### 4.3. Choice of Tracked Surgical Instrument

Ideally, the ability to track multiple or all surgical instruments would minimize timeframes during surgery without tool-tip positions providing a more complete picture of the course of resection. However, if a limited number of instrument arrays and geometries are programmed into and recognized by the neuronavigation system, the choice of surgical instrument(s) for tracking is best made in consultation with the surgeon. While the general principles of glioma resection do not vary, each surgeon may rely on different primary instruments based on their individual preferences, operating styles, training, and tumor characteristics. The suction catheter tends to be the most ubiquitously used instrument throughout the duration of the surgery. However, its shape and angle hinder line-of-sight when a stock tracking adapter array is mounted on it. The bipolar forceps, in our experience, was the second most commonly used instrument relative to the surgical duration. In some cases, particularly low-grade gliomas, we noted a preference for the Cavitron ultrasonic surgical aspirator (CUSA).

### 4.4. Instrument Tracking Arrays

The size ‘M’/’ML’ instrument adapter arrays from Brainlab made of stainless steel facilitate convenient sterilization but add considerable weight to the instrument being tracked and likely inconvenience the surgeon. Considering the benefit our surgeons perceived from our technique, they did not mind this minor hindrance. However, to address this issue and prevent any compromise to the surgeon’s dexterity, we plan to 3D print instrument adapter arrays from lighter materials and design them such that their configurations enable tracking of instruments such as suction catheters which typically angle away from the camera sensor. Moreover, using lighter materials would give us the liberty to experiment with tracking arrays of larger sizes within the ambit of ergonomics and without making the instruments unduly heavy, as the ease of resolving tracking array geometries for any optical neuronavigation system would be directly proportional to their size.

### 4.5. User Interface

The current version of our module provides a static view of the 3D and 2D panels (Video S1). A researcher manually scrolls through the grayscale preoperative MRI with an overlaid 2D resection map, compares the images to the clinical neuronavigation display, and helps the surgeon locate the orthogonal MRI slices (axial, coronal, sagittal) corresponding to the region of active resection. Updates to the 3D model generated from the tool-tip positions are also manual, requiring 2–3 s each time. In the next iteration, we plan to automate these intermittent updates to the 3D model and explore an alternate view that shows the predicted 3D residual tumor as a subtraction of the tool-tip positions from the preoperative tumor model. We also intend to leverage the Volume Reslice Driver built into 3D Slicer to generate in-line MRI views that automatically reslice to the plane and location of the active tool-tip.

### 4.6. Brain Shift

**Figure 5:**
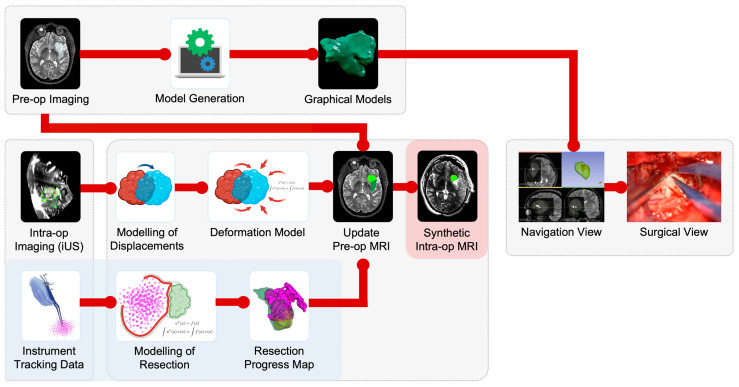
Workflow to model deformation and compensate for brain shift, utilizing preoperative MRI, intraoperative ultrasound (iUS), and surgical instrument tool-tip positions (blue panel) as source data to generate a synthetic (or virtual) intraoperative MRI.

Surgeons noted that with line-of-sight maintained, any offset between the generated resection map and the preoperative tumor model qualitatively correlated in direction and magnitude to the intraoperatively perceived brain shift. As illustrated in Figure 4 Panel C, brain shift can cause both an underestimation and overestimation of residual tumor due to False Positive Tracking and False Negative Tracking, respectively. False Negative Tracking was particularly prominent in cases where the resection cavity collapsed on itself. Our technique was originally intended only to improve and supplement deformation models derived from iUS and not as a standalone resection guidance tool (Figure 5), and we currently have ongoing research projects to estimate and compensate for brain shift using iUS. However, despite its inability to compensate for brain shift out-of-the-box, surgeons derived a clear benefit from our method because it directed them to inspect and address locations potentially harboring residual tumor. They found the system to be particularly useful for low-grade gliomas, which tend to be visually very difficult to distinguish from the healthy brain and may have radiologically diffuse boundaries with irregular shapes. Surgeons also found the system to be useful with recurrent tumors because it helped them ensure coverage at cavity margins of prior resections. These margins can be difficult to visualize directly because they often have concavities with an overhanging edge in the surgeon’s line-of-sight.

Our ongoing research investigates the use of iUS acquired at multiple surgical timepoints for MRI to iUS and iUS to iUS registration to model and compensate for brain shift [10]. Brain shift compensation could be used to correct tool-tip locations in our raw tracking data so we can more accurately estimate residual tumor. Furthermore, it would be interesting to explore the incorporation of an uncertainty map when displaying the tool-tip positions corrected for brain shift. This would allow the surgeon to interpret the degree of confidence with which they could rely on these corrected tool-tip positions [24].

### 4.7. Extrapolation to Other Pathologies and Subspecialties

Past publications [22,23] restricted their cohorts to low-grade gliomas. Woerdeman et al. [25] employed resection progress mapping to study instrument movement rather than estimate residual tumor but were the only other group to expand their cohort to high-grade gliomas, resection of epileptic foci, and skull base meningiomas. As seen in Table 2, our patient population spanned all grades of gliomas and also included resection of metastases.

Our technique can be extrapolated to other brain tumor pathologies beyond gliomas and metastases and to other neurosurgical and general surgical specialties. In fact, subspecialties such as skull base neurosurgery, which are unlikely to suffer from brain shift, could benefit from accurate residual tumor estimation using the current implementation of our module.

### 4.8. Neurosurgical Education

An additional component of the tool-tip data collected by our module is the timestamp, which can be further investigated. Our module is able to precisely log the time of each instrument position point in space. We have demonstrated how these points can be color-coded on a timescale to depict the sequence of resection or to categorize different stages of tumor resection [10]. This visualization could be used in neurosurgical education to teach resection strategies and to simulate/replay the surgery postoperatively for evaluation.

### 4.9. Alternative to iMRI

The availability of iMRI within the AMIGO Suite allows us to validate the accuracy of residual tumor estimation by our methodology against the ‘gold’ standard. iMRI access is not only limited in underserved regions of the world but also in most healthcare systems within the United States, which places patients at a tangible disadvantage. By refining our technique to comparable accuracy at low/no cost, we hope to bridge this resource gap and provide an alternative to iMRI to help achieve equitable outcomes for brain tumor patients worldwide.

### 4.10. Advancements from Prior Studies

Woerdeman et al. [25] tracked surgical instruments to study instrument movement rather than to estimate residual tumor. Hong et al. in 2007 used a method similar to ours in a cohort of five patients with gliomas. They had promising results in one prospective case and disappointing results in four retrospectively analyzed datasets. They ascribed this primarily to line-of-sight interruptions leading to an insufficient number of recorded points. Their model construction method had considerable difficulty with concave and irregularly shaped tumors [23].

Yamada et al. addressed these shortcomings and performed a quantitative estimation of residual tumor from a retrospective navigation log for tool-tip positions of a tracked bipolar forceps. Their cohort comprised 25 patients and was limited to WHO Grade II and III gliomas in the frontal lobe. Their study achieved a sensitivity of 81.8%, a specificity of 92.9%, and a positive predictive value of 72% compared to the residual tumor volumes segmented on post-resection MRIs [22]. However, unlike our sub-millimeter resolution models, their resection maps were low-resolution binary images (2 mm^3^ voxels), and they excluded regions of estimated residual tumor of smaller than 6 mm^3^. They evaluated their method by comparing volumes of residual tumor estimated from both methods, which does not measure how well the resection map spatially covers and overlaps with the actual tumor [10].

With respect to temporal performance, Hong et al. [23] reported a sampling rate of 1.5 Hz (or 3 points every 2 seconds), and Yamada et al. [22] reported a rate of 20 points/second. In our experiments, we achieved a maximum rate of 30 points/second when line-of-sight was maintained. Our processing time to refresh the 3D model of resection progress was a maximum of 3 seconds and to update the 2D map of the resection progress was between 30–50 milliseconds [10].

## 5. Conclusions

Through the largest study of prospectively logged tool-tip positions for surgical instruments in the resection of brain tumors, we demonstrate the qualitative and quantitative benefits of our generalizable, open-source tool in providing surgeons with a real-time map of their resection progress to help identify areas of potential residual tumor. Our novel technique functions parallel to standard-of-care neuronavigation without disrupting the surgical workflow and utilizes a data stream that is otherwise unused. Building upon prior experiences of our own and others [10,22,23], we have achieved a sub-millimeter spatial resolution and the highest sampling rate reported in the literature [10], offering both spatial and temporal precision. When preconditions such as line-of-sight are maintained, and brain shift is minimal, our results report qualitative and quantitative benefits in estimating residual tumor. We also explore, in detail, factors that can underestimate or overestimate residual tumor, and suggest refinements to the workflow to minimize these effects. In most cases, these shortcomings can be addressed by combining them with other low-cost modalities such as iUS. Beyond its standalone utility, we intend to integrate this into our ongoing work that uses iUS to monitor and model brain shift (Figure 5). In future efforts, we will also focus on improving the calibration accuracy for the tracked surgical instruments, the ergonomics of the instrument adapter arrays (Section 4.4) and automating aspects of the user interface (Section 4.5). We also plan to add a visualization component of the time sequence and the ability to examine tracking during selected time intervals, which will facilitate review from the standpoint of improving surgical efficiency while also serving as an educational tool. Based on the overwhelmingly positive feedback from our surgeons about the level of resection guidance offered by our module in its current iteration, we also foresee it as a low/no-cost alternative to iMRI for underserved regions of the world.

## Figures and Tables

**Table 1:** Distribution of patient demographics and intracranial pathologies (*n* = 42).

Variables	Median [IQR], Mean ± SD, % (*n*)
**Demographics**	
Age	46 [31–61]
Sex	
*Female*	*47.6% (20)*
*Male*	*52.4% (22)*
Race	
*White*	*88.1% (37)*
*Asian*	*7.1% (3)*
*African American*	*2.4% (1)*
**Pathology**	
Gliomas	84% (35)
*Glioblastoma*	*26.4% (11)*
*Astrocytoma*	*19.2% (8)*
*Oligodendroglioma*	*14.4% (6)*
*Anaplastic Oligodendroglioma*	*12.0% (5)*
*Anaplastic Astrocytoma*	*4.8% (2)*
*Others*	*7.2% (3)*
Metastases	14.4% (6)
Other	2.4% (1)

**Table 2:** Distribution of tumor laterality, anatomical compartments, and intraoperative opening of cerebrospinal compartments (*n* = 44).

Variables	Median [IQR], Mean ± SD, % (*n*)
**Preoperative MRI**	
Laterality	
*Left*	*50.0% (22)*
*Right*	*45.5% (20)*
*Bilateral*	*4.5% (2)*
Previous cranial surgery	
*Yes*	*65.9% (29)*
*No*	*34.1% (15)*
Resection cavity	
*Yes*	*40.9% (18)*
*No*	*59.1% (26)*
Anatomic compartments	
*Frontal*	*56.8% (25)*
*Temporal*	*52.3% (23)*
*Insular*	*27.3% (12)*
*Limbic*	*20.5% (9)*
*Parietal*	*13.6% (6)*
*Occipital*	*4.5% (2)*
*Basal ganglia*	*4.5% (2)*
Components	
*Cystic*	*36.4% (16)*
*Enhancing*	*56.8% (25)*
*Non-enhancing*	*77.3% (34)*
Eloquence	
*Yes*	*63.6% (28)*
*No*	*36.4% (16)*
**Intraoperative findings and iMRI**	
Opening of ventricle	
*Yes*	*38.6% (17)*
*No*	*61.4% (27)*
Opening of basal cisterns	
*Yes*	*34.1% (15)*
*No*	*65.9% (29)*
Brain shift	
*Yes*	*63.6% (28)*
*No*	*36.4% (16)*
Gross total resection	
*Yes*	*22.7% (10)*
*No*	*77.3% (34)*

Eloquence was determined anatomically by cerebral structures with readily identifiable neurological function in which injury results in disability or areas with significant fMRI activations on the tumor [18].

**Table 3:** Summary of measurements of the volume of the tumor segmented from preoperative MRI, the volume of the residual tumor segmented from iMRI, the % of the preoperative tumor resected at the time of iMRI, the % of the preoperative tumor covered by the tracked resection cavity (True Positive Tracking), and the % of the residual tumor covered by the tracked resection cavity (False Positive Tracking). For the complete set of measurements, see Appendix A, Table A1.

	Preoperative Tumor Volume (cm^3^)	Residual Tumor Volume (cm^3^)	Tumor Resected Based on iMRI (%)	Tumor Resected Based on Tool-Tip Tracking (%)	False Positive Tracking (%)
Mean	21.1	3.9	79.1	61.5	16.2
Median	16.4	2.9	86.7	60.4	12.6
Minimum	0.1	0.0	7.7	15.2	1.5
Maximum	86.2	18.2	100.0	99.7	47.9

## Data Availability

The raw data supporting the conclusions of this article can be made available by the authors upon request. The custom module for 3D Slicer created by Sarah Frisken to track time-stamped tool-tip positions of surgical instruments in this project can be downloaded from the following GitHub repository: https://github.com/sarahfrisken/continuous-monitoring-tracking.

## Supplementary Materials

The following supporting information can be downloaded at: https://www.mdpi.com/article/10.3390/cancers15030825/s1, Video S1 shows a view of the Brainlab neuronavigation system while actively navigating with a tracked bipolar forceps (top left), a corresponding view of the surgical field through the operating microscope (bottom left) with the bipolar forceps in blue, and a ‘Four-up’ view of 3D Slicer (right) with three panels showing orthogonal 2D overlays (axial—red slice, sagittal—yellow slice, coronal—green slice) recording corresponding tool-tip positions of the bipolar forceps as white voxels on a grayscale preoperative MRI background and a fourth panel showing the tool-tip positions populating as 3D shapes within the preoperative tumor model (green) to indicate resected regions.

## Appendix A

**Table A1:** Measurements of the volume of the lesion from preoperative MRI, the volume of the residual tumor from iMRI, the % of the lesion resected at the time of iMRI, the % of the lesion covered by the tracked resection cavity (True Positive Tracking), and the % of the residual covered by the tracked resection cavity (False Positive Tracking).

Case ID	Preoperative Tumor Volume (cm^3^)	Residual Tumor Volume (cm^3^)	Tumor Resected Based on iMRI (%)	Tumor Resected Based on Tool-Tip Tracking (%)	False Positive Tracking (%)
1	33.5	14.7	56.1	46.5	3.5
2	36.8	3.1	91.6	60.1	16.6
3	13.0	4.4	65.7	63.7	6.25
4	38.1	0.3	99.3	57.6	3.1
5	6.3	2.5	60.0	50.1	14.6
6	6.5	3.7	42.6	46.2	5.8
7	35.3	12.3	65.1	50.8	7.3
8	1.8	0.0	100.0	40.25	0
9	4.9	0.0	100.0	15.22	0
10	8.6	5.1	40.2	66.5	6.3
11	6.3	0.2	97.3	72.5	4.4
12	28.9	3.8	86.7	55.4	12.6
13	8.7	2.5	71.1	52.1	1.5
14	28.9	2.9	89.9	41.5	10.3
15	16.4	2.8	82.8	62.3	30.4
16	48.1	8.6	82.1	60.4	7.7
17	9.4	3.2	66.3	83.5	47.9
18	86.2	2.4	97.2	72.8	42.9
19	10.5	0.4	96.1	69.5	10.1
20	38.0	4.5	88.1	64.2	33.2
21	11.9	6.0	50.0	68.6	37.9
22	0.8	0.0	100.0	99.7	0
23	0.8	0.0	100.0	44.7	0
24	0.1	0.0	100.0	98	0
25	35.5	0.0	100.0	78.4	0
26	19.7	0.0	100.0	85.3	0
27	30.3	0.6	97.9	67.7	17.5
28	16.4	9.4	42.9	56.3	6.8
29	7.7	0.3	96.0	55.6	38.7
30	40.9	6.4	84.4	50.3	10.9
31	14.7	3.7	75.1	75.2	3.9
32	57.9	2.0	96.5	69	40.4
33	20.4	9.7	52.6	58.3	13.5
34	4.7	0.0	100.0	69.8	0
35	37.0	1.1	97.0	41.3	1.5
36	18.4	10.1	45.0	56.5	13.5
37	9.9	0.0	100.0	71.2	0
38	19.8	18.2	7.7	45.7	7
39	17.5	5.3	69.9	78	14.8
40	8.2	0.0	100.0	53.1	0
41	11.8	5.3	55.0	76.4	29
42	23.5	7.5	68.1	84.5	13
43	27.2	4.5	83.6	64.4	21.1
44	42.8	0.0	100.0	44.7	0

**Table A2:** Distribution of clinical and surgical descriptors and metadata for all cases (R: right, L: left, B: bilateral).

Case ID	Age/Sex	Race	Laterality, Anatomic Compartments		Eloquence	Cystic	Enhancing	Non-enhancing	Ventricle Opened?	Cistern Opened?	Brain Shift	GTR	Residual Location	Pathology	WHO Grade	MIB-1 Index	MGMT Promoter	IDH Mutation	1p/19q Status
1	43/F	White	R	Temporal, Mesial Temporal, Thalamic	Yes	No	No	Yes	Yes	Yes	Yes	No	Thalamus	Glioblastoma	4	5.46%	Methylated	-	Retained
2	26/M	White	L	Frontal, Insular, Temporal	Yes	No	No	Yes	No	No	Yes	No	Superior, Frontal	Anaplastic Astrocytoma	3	5%	Partially Methylated	+	Retained
3	38/M	White	R	Insular	No	No	No	Yes	No	Yes	Yes	No	Anterior, Posterior	Diffuse Astrocytoma	2	2%	Unmethylated	+	Retained
4	62/M	White	R	Frontal, Insular, Temporal	Yes	Yes	Yes	No	No	Yes	Yes	Yes	None	Glioblastoma	4		Unmethylated	-	Retained
5	42/M	White	R	Frontal, Insular, Temporal	Yes	No	No	Yes	No	No	No	No	Medial, Anterior, Superior	Oligodendroglioma	2	1%	Methylated	+	Co-deleted
6	52/F	White	L	Frontal, SMA	Yes	No	No	Yes	No	No	No	No	Anterior, Lateral, Inferior, Posterior	Glioblastoma	4	>80%	Unmethylated	-	Pending
7	28/M	White	L	Mesial Temporal	Yes	No	No	Yes	Yes	No	No	No	Superior, Posterior, Medial, Anterior	Anaplastic Astrocytoma	3	10%	Methylated	+	Retained
8	45/F	White	R	Frontal, Parietal	Yes	Yes	No	Yes	No	No	Yes	Yes	None	Oligodendroglioma	2	0.68%	Methylated	+	Co-deleted
9	58/M	White	B	Multifocal (Frontal, Parietal, Occipital)	Yes	Yes	Yes	No	No	No	Yes	No	Right Basal Frontal, Right Frontal, Right Occipital	Metastatic Carcinoma from Lung Primary					
10	49/F	White	B	Frontal	No	Yes	Yes	No	Yes	No	Yes	No	Anterior, Medial	Anaplastic Oligodendroglioma	3	3.70%	Unknown	+	Co-deleted
11	33/F	White	L	Parietal	No	Yes	Yes	Yes	Yes	No	Yes	Yes	None	Low-grade Glioma		7%	Methylated	-	Retained
12	59/F	White	L	Temporal, Mesial Temporal, Thalamic	Yes	Yes	Yes	Yes	Yes	Yes	Yes	No	Medial, Posterior	Glioblastoma	4		Unmethylated	-	Retained
13	61/F	White	L	Temporal, Mesial Temporal, Capsular	Yes	Yes	Yes	Yes	Yes	Yes	Yes	No	Temporal, Internal Capsule	Glioblastoma	4	30%	Unmethylated	-	Retained
14	24/M	White	L	Temporal, Insular	Yes	Yes	Yes	Yes	Yes	Yes	Yes	No	Medial, Lateral, Posterior, Inferior	Low-grade Glioma/Glioneural Tumor	2	10%		-	Retained
15	64/F	White	R	Frontal, Parietal	Yes	Yes	No	Yes	No	No	Yes	No	Anterior, Medial, Inferior	Anaplastic Oligodendroglioma	3	30%	Methylated	+	Co-deleted
16	25/M	White	R	Frontal, Insular, Temporal	Yes	No	Yes	Yes	Yes	Yes	Yes	No	Insula, Amygdala, Frontal	Glioblastoma	4	15%	Unmethylated	+	Retained
17	53/F	White	L	Frontal, SMA	Yes	No	No	Yes	No	No	No	No	Posterior, Lateral, Inferior, Anterior	Glioblastoma	4	>80%	Unmethylated	-	Retained
18	51/M	White	L	Frontal, Temporal	Yes	No	Yes	Yes	No	Yes	Yes	No	Frontal Operculum	Anaplastic Oligodendroglioma	3		Methylated	+	Co-deleted
19	30/M	Asian	L	Temporal	Yes	No	No	Yes	No	No	Yes	Yes	None	Oligodendroglioma	2	10%	Methylated	+	Co-deleted
20	52/F	White	R	Frontal, Temporal	Yes	Yes	Yes	No	Yes	Yes	No	Yes	None	Metastatic Breast Carcinoma					
21	36/F	White	R	Occipital	Yes	No	No	Yes	No	No	No	No	Lateral	Oligodendroglioma	2	4%	Methylated	+	Co-deleted
22	61/M	White	R	Frontal, SMA	Yes	No	Yes	Yes	No	No	No	No	Lateral	Anaplastic Oligodendroglioma	3	<2%	Methylated	+	Co-deleted
23	40/M	White	L	Frontal	No	No	Yes	Yes	No	No	No	Yes	None	Astrocytoma	4	<3%	Methylated	+	Retained
24	43/F	White	R	Temporal	No	No	Yes	No	No	No	No	No	Medial	Abnormally Thickened Blood Vessels and Chronic Inflammatory Infiltrate					
25, 26	72/M	White	R	Temporal	No	No	Yes	Yes	No	No	No	No	Anterior	Glioblastoma	4		Methylated	-	Retained
27	63/F	White	R	Frontal	Yes	Yes	Yes	Yes	No	No	Yes	No	Posterior, Medial	Anaplastic Oligodendroglioma	3	16%	Methylated	+	Co-deleted
28	50/M	White	L	Temporal, Mesial Temporal, Insular	Yes	Yes	Yes	Yes	Yes	Yes	Yes	No	Insula	Glioblastoma	4		Unmethylated	-	Retained
29	70/M	White	L	Frontal	No	No	Yes	No	No	No	Yes	No	Posterior	Metastatic Melanoma					
30	46/F	White	R	Thalamus, Caudate	Yes	No	No	Yes	Yes	No	Yes	No	Inferolateral Thalamus	Glioblastoma	4	5%	Methylated	-	
31	45/M	White	L	Frontal, Insular, Temporal, Mesial Temporal	Yes	No	No	Yes	Yes	Yes	Yes	No	Temporal, Insula	Astrocytoma	3	2%	Unmethylated	+	Retained
32	24/F	Asian	R	Frontal, SMA	Yes	Yes	Yes	Yes	No	No	Yes	No	Posterior, Inferior	Astrocytoma	4		Methylated	+	Retained
33	26/F	Declined	R	Temporal, Insular	Yes	Yes	No	Yes	Yes	No	Yes	No	Insula, Temporal	Oligodendroglioma	2	2%	Partially Methylated	+	Co-deleted
34	31/F	White	L	Frontal	No	No	No	Yes	No	No	No	No	Left Frontal Medial and Inferior	Astrocytoma	3	15%	Partially Methylated	+	Retained
35	66/F	White	L	Frontal, Temporal	No	No	Yes	No	Yes	No	Yes	Yes	None	Metastatic Poorly Differentiated Carcinoma					
36	62/M	White	L	Temporal, Mesial Temporal	Yes	No	No	Yes	Yes	Yes	Yes	No	Temporal, Amygdala, Hippocampus, Insula	Diffuse Glioma	>2	2-3%	Partially Methylated	-	Retained
37	32/F	African American	L	Parietal	Yes	No	No	Yes	No	No	No	Yes	None	Astrocytoma	2	2%	Methylated	+	Retained
38	69/M	White	L	Frontal, Insular, Mesial Temporal	Yes	No	Yes	Yes	No	Yes	Yes	No	Amygdala, Anterior and Posterior insula	Glioblastoma	4		Methylated	-	Retained
39	66/F	White	R	Frontal, SMA	Yes	No	Yes	No	No	No	No	No	Anterior, Medial, Posterior, Inferior	Glioblastoma	4	>50%	Unmethylated	-	Retained
40	43/M	Asian	R	Frontal, Insular, Temporal	Yes	No	No	Yes	No	No	No	Yes	None	Astrocytoma	2	3%	Methylated	+	Retained
41	60/M	White	L	Frontal	No	Yes	Yes	No	No	No	Yes	Yes	None	Metastatic Melanoma					
42	30/M	White	R	Temporal, Insular	Yes	No	Yes	Yes	Yes	Yes	No	No	Posterior, Anterior	Astrocytoma	2	5-10%	Unmethylated	+	Retained
43	42/F	White	L	Frontal, Insular, Temporal	Yes	No	No	Yes	Yes	Yes	Yes	No	Small Segment of Anterior Superior Insular Gyrus, Segment of the Posterior Insula and the Posterior Superior Temporal Segment of the tumor	Oligodendroglioma	2	5%	Methylated	+	
44	70/M	White	L	Parietal, Temporal	Yes	Yes	Yes	No	No	No	Yes	No	Posterior, Inferior	Metastatic Gastrointestinal Stromal Tumor					
